# Notch3 restricts metastasis of breast cancers through regulation of the JAK/STAT5A signaling pathway

**DOI:** 10.1186/s12885-023-11746-w

**Published:** 2023-12-20

**Authors:** Min-Na Chen, Ze-Xuan Fang, Zheng Wu, Jing-Wen Bai, Rong-Hui Li, Xiao-Fen Wen, Guo-Jun Zhang, Jing Liu

**Affiliations:** 1 Department of Medical Oncology, Cancer Hospital of Shantou University Medical College, Shantou, China; 2 The Breast Center, Cancer Hospital of Shantou University Medical College, Shantou, China; 3 Department of Medical Oncology/Xiamen Key Laboratory for Endocrine-Related Cancer Precision Medicine, Xiamen University Medical School, Xiang’an Hospital of Xiamen University, Xiamen, China; 4 Xiamen Key Laboratory for Endocrine-Related Cancer Precision Medicine/Department of Breast and Thyroid Surgery, Xiamen University Medical School, Xiang’an Hospital of Xiamen University, Xiamen, China

**Keywords:** Notch3, STAT5A, Breast cancer, Metastasis, Transcription

## Abstract

**Purpose:**

To explore the potential role of signal transducer and activator of transcription 5A (STAT5A) in the metastasis of breast cancer, and its mechanism of regulation underlying.

**Methods and results:**

TCGA datasets were used to evaluate the expression of STAT5A in normal and different cancerous tissues through TIMER2.0, indicating that STAT5A level was decreased in breast cancer tissues compared with normal ones. Gene Set Enrichment Analysis predicted that STAT5A was associated with the activation of immune cells and cell cycle process. We further demonstrated that the infiltration of immune cells was positively associated with STAT5A level. Influorescence staining revealed the expression and distribution of F-actin was regulated by STAT5A, while colony formation assay, wound healing and transwell assays predicted the inhibitory role of STAT5A in the colony formation, migratory and invasive abilities in breast cancer cells. In addition, overexpression of the Notch3 intracellular domain (N3ICD), the active form of Notch3, resulted in the increased expression of STAT5A. Conversely, silencing of Notch3 expression by siNotch3 decreased STAT5A expression, supporting that STAT5A expression is positively associated with Notch3 in human breast cancer cell lines and breast cancer tissues. Mechanistically, chromatin immunoprecipitation showed that Notch3 was directly bound to the STAT5A promoter and induced the expression of STAT5A. Moreover, overexpressing STAT5A partially reversed the enhanced mobility of breast cancer cells following Notch3 silencing. Low expression of Notch3 and STAT5A predicted poorer prognosis of patients with breast cancer.

**Conclusion:**

The present study demonstrates that Notch3 inhibits metastasis in breast cancer through inducing transcriptionally STAT5A, which was associated with tumor-infiltrating immune cells, providing a novel strategy to treat breast cancer.

**Supplementary Information:**

The online version contains supplementary material available at 10.1186/s12885-023-11746-w.

## Introduction

Breast cancer affects the health of millions of women in China and worldwide annually [1]. Although great progress has been made in breast cancer research during the past decades, recurrence and metastasis remain high, largely due to breast cancer being a heterogeneous disease characterized by different molecular drivers [2, 3].

Epithelial-mesenchymal transition (EMT) is a critical biological process during normal embryonic development and disease. In particular, EMT in breast cancer endows malignant epithelial tumor cells with increased motility and invasiveness, related to metastasis, chemoresistance, and/or radio-resistance [4, 5]. Previous research suggests EMT is the probable first key step in cancer development and involves altered biological signaling pathways, cancer stem cells, and ncRNAs [6, 7]. However, although Notch [8], Wnt [9], and GATA [10] signaling pathways are reported to be involved in the malignant transformation of mammary epithelia, causing abnormal cell growth and differentiation, the complex mechanism of breast cancer metastasis still needs further investigation.

Signal transducer and activator of transcription 5A (STAT5A), originally identified in mammary glands and activated by prolactin [11], is reported to be the principal and obligate mediator of mammopoietic and lactogenic signaling [12, 13]. It is found that prolactin-directed mammary epithelial cell differentiation program related to STAT5A offer a good prognosis in human breast cancer [14, 15]. However, in mouse breast cancer, Stat5a transcriptional increases the secretion of IL-1ra, a traditional immune-suppressive cytokine, which was the downstream of miR-100 to maintain phenotype of tumor-associated macrophages and promote tumor metastasis [16]. Meanwhile, STAT5A has also been found to be an inducer of chemoresistance and facilitate the resistance of breast cancer to doxorubicin through regulating ABCB1 [17]. Moreover, downregulation of prolactin-mediated JAK2/STAT5A signaling by Mucuna pruriens, an ancient Indian medicinal plant enhanced cisplatin efficacy in the treatment of breast cancer cells [18]. The above findings indicate different effects of STAT5A on breast cancer through diverse mechanisms. It is curious to investigate the regulatory mechanism of abnormal STAT5A in human breast cancer patients and its potential function.

The conserved Notch signaling pathways play critical roles in embryonic development and cellular self-renewal [19]. Importantly, diverse Notch proteins perform tumor suppressor and/or oncogenic activities in hematological malignancies and several solid tumors, including breast carcinoma [20]. Among different Notch family members, Notch3 is found to maintain a luminal phenotype and suppress the tumorigenesis and metastasis of breast cancer, in part involving estrogen receptor-α (ERα) serving as a transcriptional target of Notch3 [21]. Notch3 exerts its biological effects by regulating target gene expression via interaction with the nuclear CBF1/RBP-Jκ/Suppressor of Hairless/LAG-1 (CSL) transcriptional complex or noncanonical expression [22]. Mis-activation and dysfunction of Notch3 are associated with the oncogenesis and development of different cancers through transcriptionally activating its downstream genes [23]. The tumor suppressor gene, *PTEN*, was reported as a Notch3 downstream target to suppress cell proliferation and tumorigenesis in breast cancer [24], while Kibra-mediated Hippo/YAP signaling was also transcriptionally activated by Notch3 in breast cancer epithelial cells to inhibit EMT process [25]. However, the molecular mechanism of Notch3 in breast cancer has not yet been completely deciphered.

In our preliminary experiments, the consistent expression pattern of STAT5A and Notch3 in different breast cancer cell lines was found and a CSL-binding site was predicted in the promoter region of STAT5A. As Notch3 and STAT5A perform similar tumor-suppressive effects on EMT and metastasis of breast cancer, current study investigated the relationship between STAT5A and Notch3 and their roles in tumorigenesis and metastasis of breast cancer. The purpose of the current study is to explore the regulatory mechanism of EMT and metastasis through a Notch3/STAT5A axis in breast cancer.

## Materials and methods

### Cell culture

Breast cancer cell lines, MDA-MB-231, BT549, MCF-7, T47D, and SKBR3 were purchased from the American Type Culture Collection (ATCC). All cells were cultured in Dulbecco’s Modified Eagle’s Medium (DMEM), containing 10% fetal bovine serum (FBS) and 1% penicillin/streptomycin, in a humidified 5% CO_2_ incubator at 37°C.

### Plasmids and small interfering RNAs (siRNAs)

The constructed clone for Notch3, pCMV-Sport6-N3ICD, and its control vector pCMV-Sport6, were gifts from Prof. Michael Wang [26]. To overexpress STAT5A in breast cancer, plasmids pCMV3-C-GFP and pCMV3-STAT5A-GFP were purchased from Sino Biological Inc. (Beijing, China). After confirming the binding site of Notch3 on the promoter region of STAT5A, the entire promoter region (-2016/ + 85) and the CSL binding sites (-574/-287) in the STAT5A promoter were inserted into the KpnI/HindIII sites of pGL3-Enhancer (Promega) to engineer STAT5A promoter-driven and CSL-driven luciferase reporter genes, respectively denoted pGL3-STAT5A-pro-luc-E and pGL3-STAT5A-23-pro-luc-E. A reporter with a deletion of the binding sites in the STAT5A promoter region was also constructed and denoted pGL3-STAT5A-M23-pro-luc-E.

Oligonucleotides for siNotch3, siSTAT5A and corresponding negative control (siNC) were purchased from GenePharma (Suzhou, China). The sequences are as follows; siNotch3#1: 5’- UAUAGGUGUUGACGCCAUCCACGCA-3’, siNotch3#2: 5’- GAGCCAAUAAGGACAUGCATT-3’, siSTAT5A: 5’- AUGGAUAUGUGAAACCACATT-3’, siNC: 5’- UUCUCCGAACGUGUCACGUTT-3’. For transfection, Lipofectamine 3000 (Life Technology, NY, USA) was used according to the manufacturer's protocol.

### Immunofluorescent staining

The cells were fixed with 4% paraformaldehyde for 15 min, permeabilized with 0.5% Triton-X-100 for 8 min. Then, 2.5% goat serum was used to block the cells for 30 min, followed with primary antibody (anti-Vimentin, 1:200 dilution) incubation overnight at 4 °C. After incubation with secondary antibody, cell were counterstained with DAPI (Invitrogen Corp.) for 3 min. ImageJ software was used to quatified the immunofluorescent intensity of Vimentin.

To assess stress fiber formation, the blocked cells were incubated with phallinium cyclic tide (Thermo Fisher Scientific, 2157163) for 30 min at room temperature in dark. After washed with PBS, the cells were stained with DAPI-containing anti-fluorescence quench agent (Solarbio, S2110) for 5 min. Then, the slides were sealed and observed under a LSM800 confocal microscope (Zeiss, Germany).

### Western blot analysis

Total proteins were extracted from whole cell lysates, resolved by 10% SDS-PAGE, and transferred to a PVDF membrane (Millipore, MA). Primary antibodies for Notch3 (5276S), E-cadherin (3195S), Vimentin (5471S), and β-actin (3700S) were purchased from Cell Signaling Technology (Danvers, USA). Anti-STAT5A (L-20) was purchased from Santa Cruz Biotechnology Inc. (Santa Cruz, USA), anti-phospho-STAT5 (Y694) (E208) was purchased from Abcam Inc. (Cambridge, USA), and anti-GAPDH (TA-08) was purchased from ZSGB-BIO (Beijing, China). A chemiluminescence system was used to detect specific proteins after incubations with corresponding secondary antibodies. Quantity One software was used to quatitify gray value analysis of Western blot results.

### Real-time PCR

Total RNA was isolated using TRIzol (Invitrogen, USA), and the synthesis of cDNA was performed using a TAKARA Synthesis Kit (D6110A, Japan) according to the manufacturer’s instructions. To detect mRNA expression, quantitative real-time PCR was carried out with SYBR Select Master Mix (Thermo Fisher, MA, USA) in a CFX96 Real-time PCR Detection System (Bio-Rad, CA, USA). To normalize the amount of mRNA in each sample, β-actin was used as an internal normalization control. The results are expressed as fold change, calculated by the ∆∆Ct method, relative to the control sample and analyzed using GraphPad software.

### Chromatin immunoprecipitation (ChIP) assay

ChIP assays were performed as previously described [21]. Cell lysates were collected from MCF-7 cells at 80% confluence in a 100-mm dish and incubated with anti-Notch3 antibody or normal rabbit IgG (CST 2729) as control. Ten percent of total cell lysate was used as input for PCR amplification. The promoter region of STAT5A contained 5 CSL-binding elements (TGGGAA), located at -32/-37, -489/-494, -548/-553, -1757/-1762, and -1927/-1932 upstream of exon 1. PCR was used to amplify the regions containing the CSL-binding elements using the primers in Table 1. A 171 bp product (located -775 to -605 bp, denoted STAT5A-NC), that did not contain the CSL-binding element, was also amplified by PCR as a negative control.

**Table 1:** Primer sequences for ChIP assay

Primers		Sequences (5' to 3')	Amplicon (bp)
Primer#1	F	CGACCTTACCAAACCCCTTG	168
	R	TGAGGCTGGTTCTACCTTCAG	
Primer#23	F	TGCCAAATCCATTGCTCA	287
	R	TCTTGCCCCTTACTACCTC	
Primer#4	F	GGGACAAACAAGGGGACTGAA	200
	R	TCTCCCTTTGAGATGCAGTGG	
Primer#5	F	TCTGATCTTCCTGAACCCCCA	184
	R	AGTCGTGGCTCCAGATAACAC	
Primer NC	F	CCACCCAAATGTGGCAATGG	170
	R	ACTGGAATGAAAGCCAAGTGC	

### Dual-luciferase reporter assays

To determine the influence of Notch3 expression on STAT5A promoter activity, pGL3-STAT5A-pro-luc-E, pGL3-STAT5A-23-pro-luc-E or pGL3-STAT5A-M23-pro-luc-E was co-transfected into BT549 cells with pCMV-sport6-N3ICD in a dose-dependent manner, as along with pRL-SV40 for internal reference. In addition, pGL3-STAT5A-pro-luc-E, pGL3-STAT5A-23-pro-luc-E or pGL3-STAT5A-M23-pro-luc-E was co-transfected into MCF-7 cells with siN3 in a dose-dependent manner, along with pRL-SV40 for internal reference. Luciferase activity was measured by using a Dual-Luciferase Reporter Assay System (Promega).

### Wound healing assays

Cells were pretreated with mitomycin C (25 mg/mL) 30 min before a scratch wound was applied. The wound was made with a 2 mm-wide pipette tip on cells plated in culture dishes at 90% confluence. After washing with PBS, cells were allowed to migrate in complete medium, and photographs were taken (× 40) at different time point for BT549 and MCF-7. An average of five random widths along the injury line was measured for quantitation.

### Transwell assays

Cell culture inserts (8 μM pore size, BD, USA) and Matrigel invasion chambers (BD, USA) were used to carry out migration and invasion assays, respectively. Transfected cells were serum-starved for 24 h, 2 × 10^4^ BT549 cells or 5 × 10^4^ MCF-7 cells in serum-free medium were inoculated in the upper chamber, and complete medium was added in the bottom chamber. Cells were stained with 0.1% crystal violet for the assays. The exact number of cells from 5 random fields in every individual well was captured and analyzed by two independent investigators.

### Clony formation assays

Cells were diluted to 0.5 × 10^4^ cells /mL and 200 µL was added to a six-well plate after thorough mixing. The six-well plates were placed into an incubator and changed the medium every 2–3 days. The cultures were terminated when macroscopic cell colonies appeared. Cells were fixed with anhydrous formaldehyde for 15 min, and stained with 0.1% crystal violet for 5 min, then, photographed and counted under a microscope.

### Notch3/STAT5A expression in cancerous cohorts in TCGA

To evaluate the expression pattern of STAT5A in different cancereous tissues and adjacent normal, the Tumor IMmune Estimation Resource (TIMER2.0) online source (http://timer.cistrome.org/) was used based on TCGA datasets. GOBO database (http://co.bmc.lu.se/gobo/gsa.pl) was used to explore the relationship between the expression level of STAT5A and the clinicopathological parameters. To explore the relationship between Notch3 and STAT5A in patients with breast cancer, the expression levels of Notch3 and STAT5A in such patients was searched and downloaded from TCGA through cBioPortal (www.cbioportal.org). The correlation analysis and correlation figure were conducted and constructed using GraphPad Prism.

### The signaling pathway involved by STAT5A in breast cancer

To explore the potential signaling pathway involved in STAT5A function, LinkedOmics (http://www.linkedomics.org/), an multi-omics data within and across 32 cancer types, was applied to perform the enrichment analysis of STAT5A based on Gene Ontology, biological pathways, network modules, among other functional categories. After finding the relationship with immune cells and their differentiation by STAT5A level, TIMER2.0 (http://timer.cistrome.org/) was conducted to analyze the potential involvement of STAT5A in tumor-infiltrating immune cells.

### Prognostic values of Notch3 and STAT5A in patients with breast cancer

To evaluate the prognostic values of Notch3 and STAT5A in patients with breast cancers, the Human Protein Atlas (HPA) (https://www.proteinatlas.org/) and Kaplan–Meier plotter (http://kmplot.com/analysis/) were used to analyze the potential effects of Notch3 and STAT5A on overall survival (OS) and recurrence-free survival (RFS) of the patients with breast cancer.

### Statistical analysis

All experiments were carried out for at least three times. Statistical analysis was performed using SPSS 16.0 (SPSS Inc., Chicago, IL, USA). Data are presented as mean ± standard error of the mean (SEM). Statistical analyses were assessed by a two-tailed Student’s *t*-test and statistical significance was indicated with a cutoff *p*-value < 0.05.

## Results

### STAT5A was decreased in breast cancer tissues compared with normal ones and related to tumor-infiltrating immune cells

**Fig. 1:**
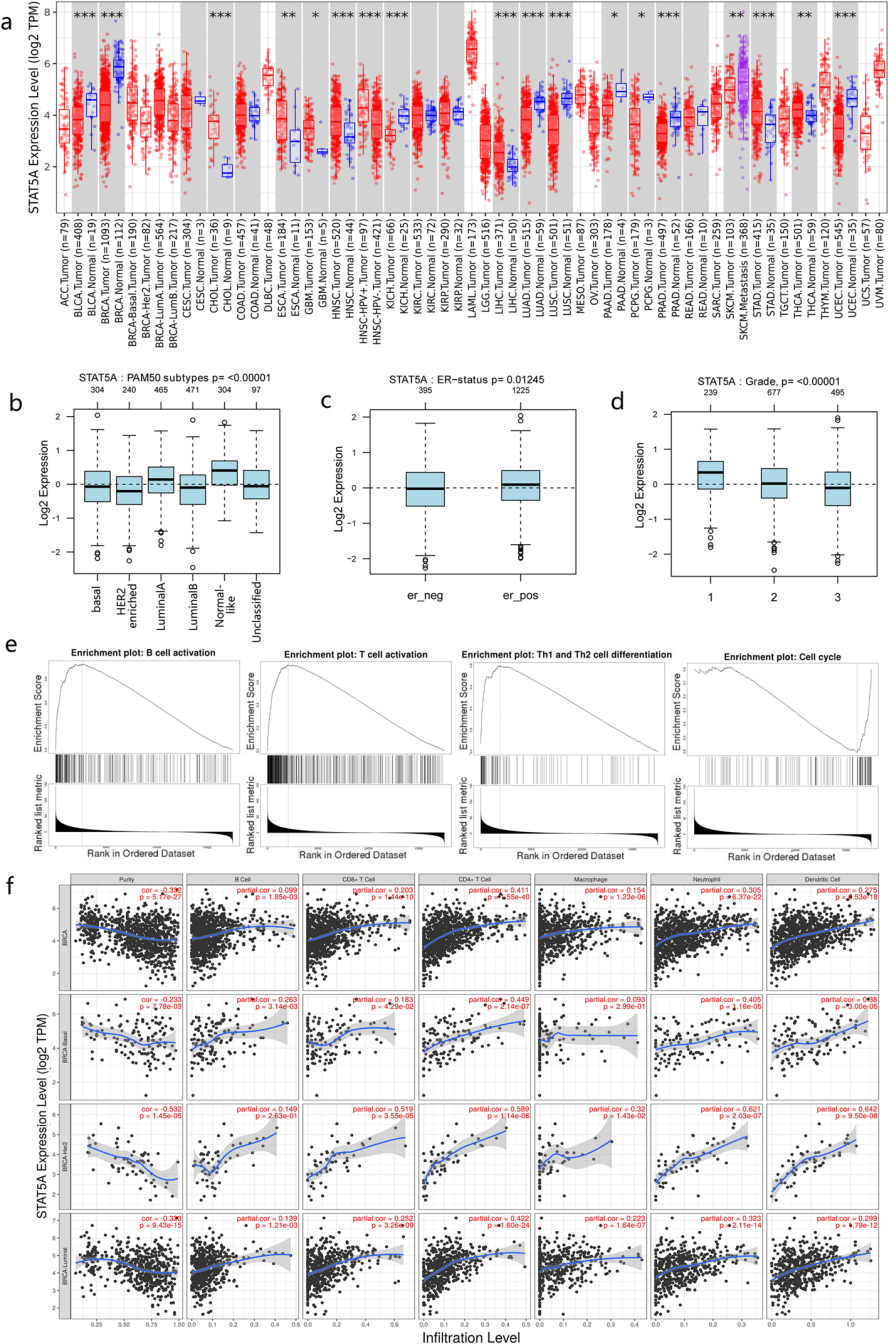
The expression of STAT5A level in breast cancer was related to tumor-infiltrating immune cells. **a** The expression of STAT5A in different types of cancer and corresponding normal tissues. * *p* < 0.05, ** *p* < 0.01, *** *p* < 0.001. **b** The expression level of STAT5A in different subtypes of breast cancer. **c** The STAT5A expression in breast cancer divided by ER status. **d** The STAT5A level in different grades of breast cancers. **e** GSEA analysis revealed the relationship between STAT5A level and B/T cell activation, as well as cell cycle. **f** The enrichment of immune cells was positively related with STAT5A level in different subtypes of breast cancers

To explore the expression pattern of STAT5A in different cancer types, TIMER2.0 was conducted and revealed that the STAT5A was decreased in cancerous tissues of bladder urothelial carcinoma (BLCA), breast invasive carcinoma (BRCA), kidney chromophobe (KICH), lung adenocarcinoma (LUAD), lung squamous cell carcinoma (LUSC), pancreatic adenocarcinoma (PAAD), pheochromocytoma and paraganglioma (PCPG), prostate adenocarcinoma (PRAD) and uterine corpus endometrial carcinoma (UCEC), compared with corresponding adjacent normal ones (Fig. 1a). Interestingly, the diverse expression of STAT5A was found in malignant tissues of cholangiocarcinoma (CHOL), esophageal carcinoma (ESCA), glioblastoma multiforme (GBM), head and neck squamous cell carcinoma (HNSC), liver hepatocellular carcinoma (LIHC), stomach adenocarcinoma (STAD), and thyroid carcinoma (THCA), which was significantly increased compared with normal (Fig. 1a).

To investigate the potential function of STAT5A in breast cancer, different expression type of STAT5A was analyzed by GOBO database. In Fig. 1b, the level of STAT5A was high in normal like and luminal A types compared with basal and HER2 enriched ones. Further analysis revealed that STAT5A level in ER + breast cancer patients was higher than that in ER- patients (*p* = 0.01245) (Fig. 1c). Importantly, when compared in different stage of breast cancer, the higher STAT5A expression level, the well differentiation of breast cancer (*p* < 0.00001) (Fig. 1d).

With potential tumor-suppressing function in breast cancer, the underlying molecular mechanism of STAT5A needs to be investigated. Used GSEA analysis methods, STAT5A level was predicted to positively associated with B/T cell activation and Th1/Th2 cell differentiation, as well as negatively related to cell cycle (Fig. 1e).

Further analysis of the different immune cells in breast cancer, the high level of STAT5A was positively related to the amount B cell, CD8 + /CD4 + T cell, Macrophage, neutrophil and dendritic cell in breast cancer, as well as in different subtypes of breast cancer (Fig. 1f), indicating the increased STAT5A has the potential to enhance tumor-related immune response.

### STAT5A inhibits EMT and metastasis of breast cancer cell lines

**Fig. 2:**
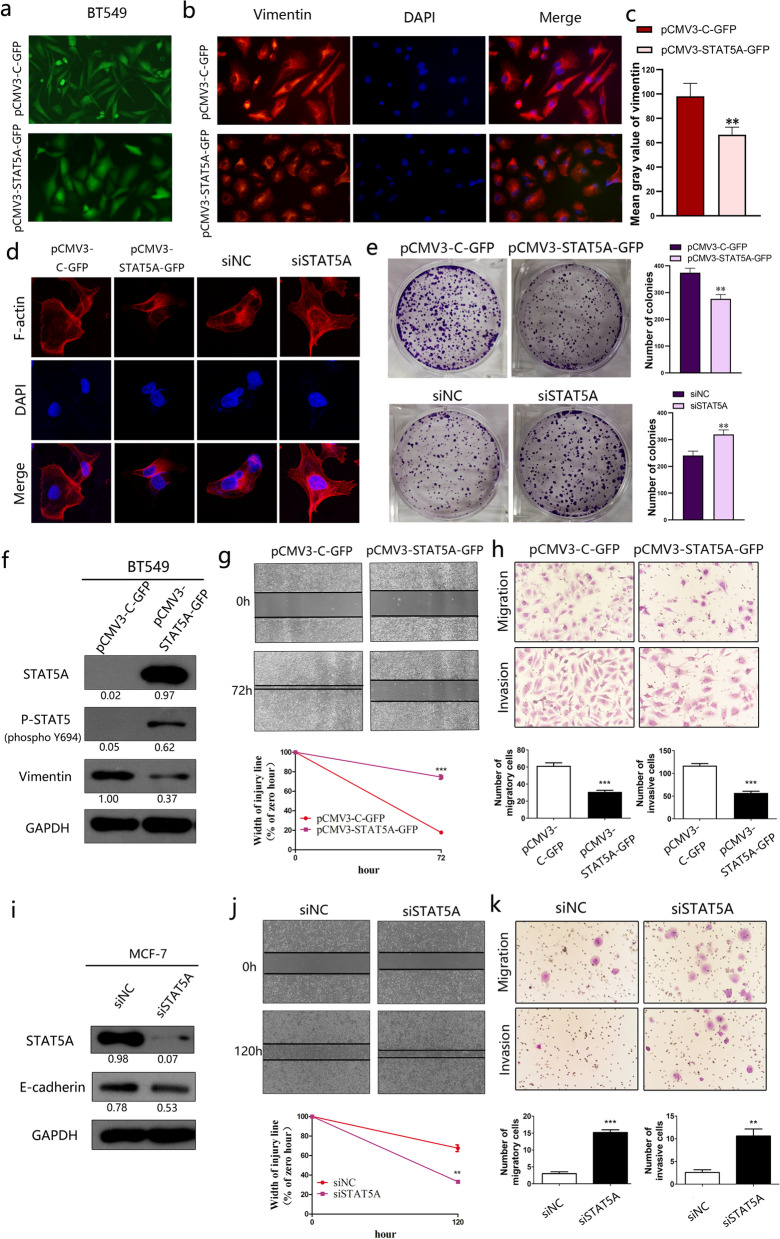
STAT5A inhibits the mobility of breast cancer cells by suppressing EMT. **a** Overexpression of STAT5A caused BT549 cells to lose their long spindle shape. **b** Immunofluorescence showing that expression of Vimentin was inhibited by STAT5A in BT549 cells. **c** ImageJ was used to quantitatively analysis the immunofluorescent staining of Vimentin. **d** Influorescence staining of F-actin in BT549 with STAT5A overexpression and MCF-7 with siSTAT5A. **e** The colony formation was suppressed by STAT5A overexpression. **f** Western blot showing in BT549 cells, increased STAT5A expression suppressed the level of the mesenchymal marker, Vimentin. **g** Wound healing of BT549 cells was suppressed by overexpressing STAT5A. **h** Invasion of BT549 was suppressed by overexpressing STAT5A. **i** In MCF-7 cells, suppression of STAT5A levels decreased the expression of the epithelial marker, E-cadherin. **j** Knockdown of STAT5A promoted wound healing of MCF-7 cells. **k** Knockdown STAT5A expression promoted invasiveness of MCF-7 cells

To determine the effect of STAT5A on breast cancer cells, pCMV3-STAT5A-GFP was used to overexpress STAT5A in the BT549 cell line. As the phenotypic switch from spindle-like to cobblestone-like morphology was consistent with the EMT proceses as well as the changed expression of epithelial and mesenchymal markers [27, 28], it is interesting to find that overexpression of STAT5A promoted the transformation of BT549 from the spindle-like mesenchymal phenotype to the cobblesone-like epithelial phenotype, compared to the controle cells (Fig. 2a).

To confirm the potential suppression of STAT5A on EMT process, the expression EMT-biomarkers and aggressive ability of breast cancers were evaluated accordingly. Immunofluorescence revealed that overexpression STAT5A reduced the expression of Vimentin, a mesenchymal marker, in BT549 cells (Fig. 2b and 2c). Meanwhile, the expression and distribution of F-actin, one of the main cytoskeletons in the cell, was suppressed by STAT5A overexpression in BT549 cells, while knockdown of STAT5A expression in MCF-7 cells promoted this process, which is related to the cellular mobility (Fig. 2d). As the main step for tumor metastasis, colony formation was also negatively related to the STAT5A expression level (Fig. 2e).

It is well known that STAT5A is phosphorylated by JAK2 to further form a p-STAT5 dimer, which is the active form of STAT5A to regulate gene expression in the nucleus [29, 30]. After transfection, expression of STAT5A and its active form, p-STAT5, were examined, which were both significantly increased (Fig. 2f). The decreased Vimentin expression was confirmed by western blotting at least 63% reduction in quantitative analysis (Fig. 2f). Conversely, knockdown of STAT5A expression with siRNA decreased the levels of E-cadherin, an epithelial marker, in MCF-7 breast cancer cells (Fig. 2i).

In wound healing assays, STAT5A overexpression suppressed cell migration compared to the control group (*p* < 0.001) (Fig. 2g), and in transwell assays, overexpression of STAT5A also suppressed both the migration and invasion of breast cancer cells compared with the control group (Fig. 2h).

Conversely, decreased expression of STAT5A enhanced the migration of MCF-7 cells in wound healing assays to recover 67% of the scratch wound distance 120 h after wounding, compared to 32% in the control group (Fig. 2j). In transwell assays, the number of breast cancer cells migrating and invading to the lower layer was increased in MCF-7 cells transfected with siSTAT5A compared with that in the negative control (Fig. 2k).

### Notch3 expression is positively associated with STAT5A levels and up-regulates the level of STAT5A and its active form (p-STAT5A) in breast cancer cells

**Fig. 3:**
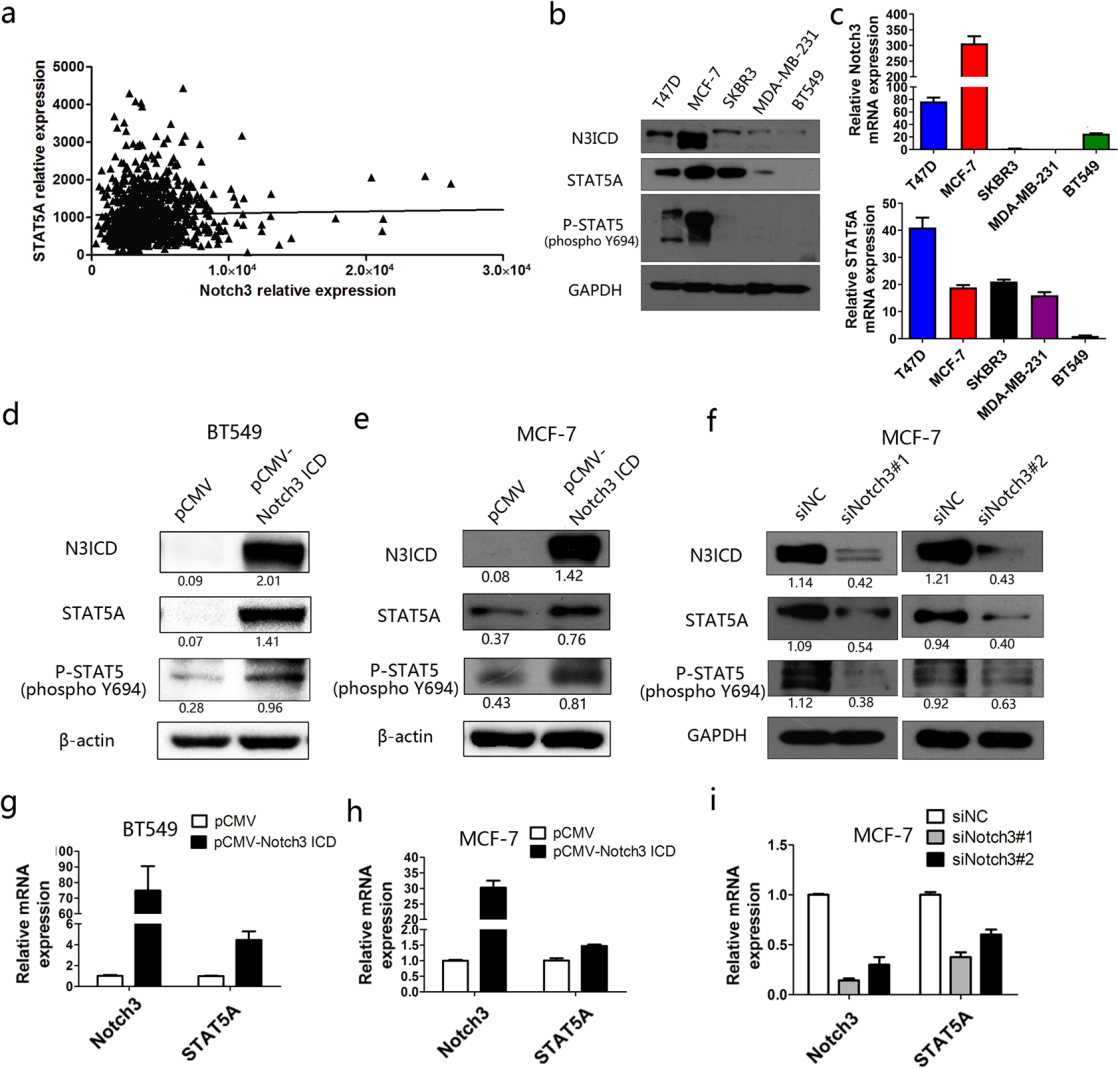
Expression of STAT5A is positively associated with Notch3 in breast cancer. **a** In TCGA (Cell 2015), the expression of STAT5A was positively associated with Notch3 in patient tissues. **b** Notch3 and STAT5A mRNA levels in different breast cancer cell lines. **c** STAT5A protein expression was positively associated with Notch3 in different breast cancer cell lines. **d** Overexpression of Notch3 promoted expression of STAT5A and its active form, p-STAT5, at the protein level in BT549 cells. **e** Overexpression of Notch3 promoted the expression of STAT5A and its active form, p-STAT5, at the protein level in MCF-7 cells. **f** In MCF-7 cells, knockdown of Notch3 decreased STAT5A and p-STAT5A at the protein level. **g** Overexpression of Notch3 increased the mRNA level of STAT5A in BT549 cells. **h** Overexpression of Notch3 increased STAT5A mRNA levels in MCF-7 cells. **i** The mRNA level of STAT5A was suppressed by siNotch3

Based on the dataset from TCGA (Cell 2015), for 817 patients with breast invasive carcinoma, the expression of Notch3 was positively associated with the levels of STAT5A (*p* = 0.001 and Spearman *r* = 0.1154, Fig. 3a). To determine the expression pattern of Notch3 and STAT5A in breast cancer cell lines, their mRNA levels, and protein levels were examined and showed that in ER-positive breast cancer cell lines (MCF-7 and T47D), Notch3 and STAT5A were both highly expressed compared with that in TNBC cell lines (MDA-MB-231 and BT549) (Fig. 3b and c). Importantly, the expression of the active form of STAT5A, p-STAT5 was also consistent with the expression pattern of Notch3 (Fig. 3b). To examine the potential effect of STAT5A, we chosed MCF-7 (with the highest STAT5A and p-STAT5 level) and BT549 (with the lowest STAT5A and p-STAT5 level) for the following experiments.

Overexpression of N3ICD upregulated the expression of STAT5A protein (Fig. 3d and e) and mRNA (Fig. 3g and h) levels. Increased Notch3 expression also promoted the phosphorylation of STAT5A (p-STAT5) (Fig. 3d and e). Conversely, knockdown of Notch3 by siRNA caused significant downregulation of STAT5A and lowered levels of p-STAT5 in breast cancer cell lines (Fig. 3f and i).

### Notch3 binds to CSL-binding elements in STAT5A promoter

**Fig. 4:**
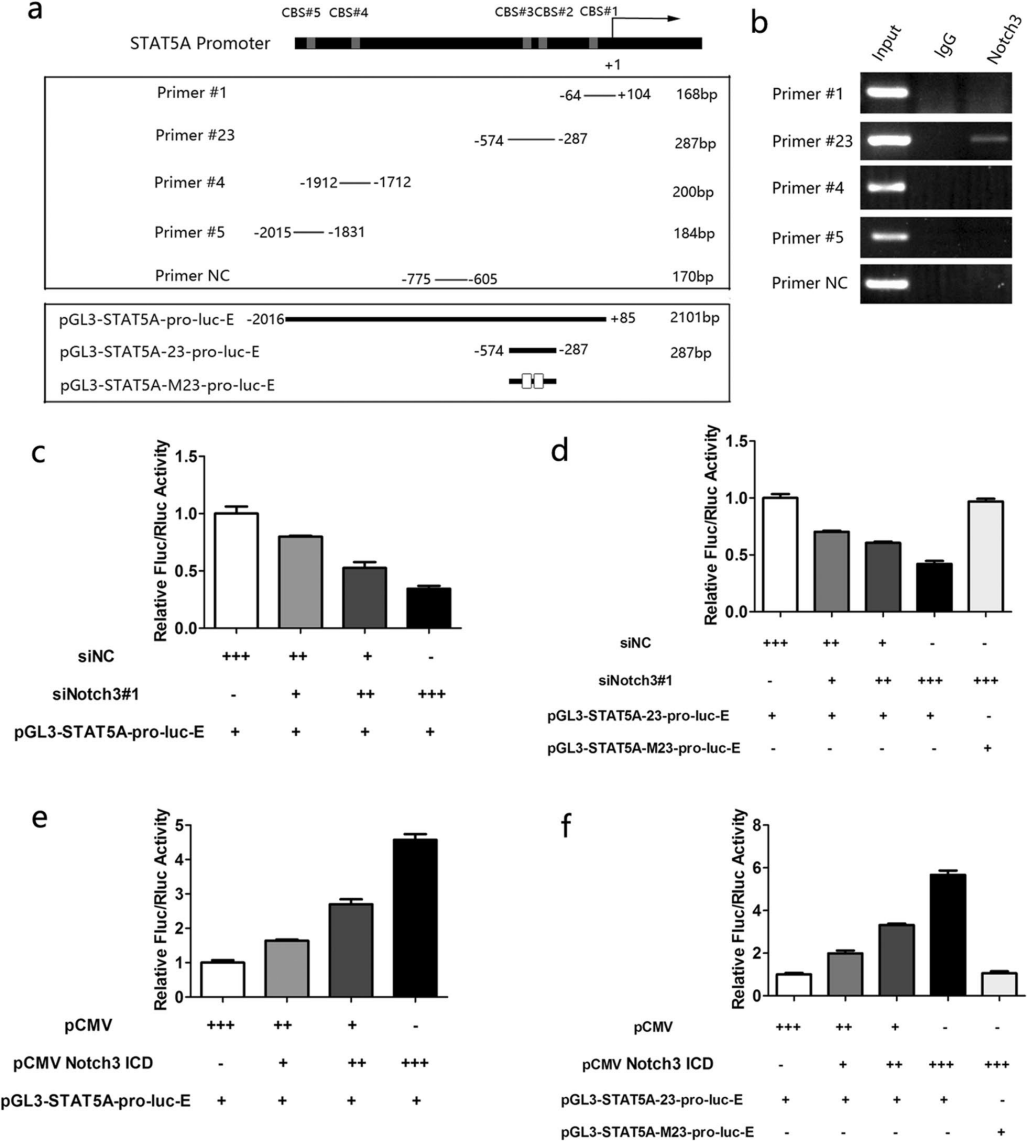
Notch3 directly binds to the STAT5A promoter and activates STAT5A expression. **a** Potential CSL binding sites of Notch3 on the STAT5A promoter, and the adjacent region, lacking Notch3 binding sites, as the negative control, and the construction strategy of luciferase reporter genes driven by wildtype and CSL-mutant STAT5A promoters. **b** ChIP results showing that Notch3 directly binds to the STAT5A-2/3 region on the promoter. **c** In MCF-7 cells, the luciferase activity of pGL3-STAT5A-pro-luc-E was suppressed by loss of Notch3 on the promoter region of STAT5A in a dose-dependent manner. **d** In MCF-7 cells, the luciferase activity of pGL3-STAT5A-pro-23-luc-E was suppressed by knockdown of Notch3 in a dose-dependent manner. The STAT5A promoter, lacking Notch3 binding sites, was not regulated by knockdown of Notch3. **e** In BT549 cells, STAT5A promoter-driven luciferase activity was activated by overexpression of Notch3 in a dose-dependent manner. **f** In BT549 cells, STAT5A promoter-driven luciferase activity was activated by the binding of Notch3 on the STAT5A promoter in a dose-dependent manner. The STAT5A promoter, lacking Notch3 binding sites, was not regulated by the overexpression of Notch3

To explore the potential regulatory mechanism of Notch3 on STAT5A through the canonical Notch signaling pathway, CSL-binding elements were searched in the promoter region of the STAT5A gene, which was downloaded from the UCSC online database (http://genome.ucsc.edu/index.html). Five potential binding sites (TGGGAA) were found at -32/-37, -489–494, -548/553, -1757/1762, and -1927/1932 upstream of exon 1 in the STAT5A promoter (Fig. 4a). As MCF-7 cells had a high-level of endogenous Notch3, the DNA binding to Notch3 was immunoprecipitated, extracted and amplified in MCF-7 cells. The ChIP results showed that Notch3 directly bound to the promoter of STAT5A, especially at the two closely linked regions 2 (-489–494) and 3 (-548/553), whereas no binding was detected in the negative control group (Fig. 4b).

### Binding of Notch3 to the STAT5A promoter upregulates the transcriptional activities of STAT5A promoter

A STAT5A promoter-driven luciferase reporter plasmid, denoted pGL3-STAT5A-pro-luc-E, was engineered by inserting the STAT5A promoter into the pGL3-Enhancer plasmid (Fig. 4a). Dual-luciferase assays found that the luciferase activity controlled by the STAT5A promoter was significantly downregulated by the knockdown of Notch3 in a dose-dependent manner in MCF-7 cells (Fig. 4c). Based on the ChIP results, the promoter region containing CSL-binding sites 2 and 3, predicted to be the active region for Notch3 transcriptional regulation, were inserted into pGL3-Enhancer to generate a Notch3-dependent reporter gene (Fig. 4a). Knockdown of Notch3 dose-dependently decreased luciferase activity for pGL3-STAT5A-pro-23-luc-E but not for reporter genes containing deletion of CSL-binding sites 2 and 3 in the STAT5A promoter (Fig. 4d). In BT549 cells, the luciferase activities of both reporters (pGL3-STAT5A-pro-luc-E and pGL3-STAT5A-23-pro-luc-E) were upregulated in a dose-dependent manner after overexpression of Notch3 (Fig. 4e and f). However, the luciferase activities did not change in the mutant reporter genes with deletion of CSL-binding sites 2 and 3 (Fig. 4f).

### Notch3-mediated suppression of EMT is attenuated by downregulating STAT5A expression

Notch3 maintains the epithelial phenotype of breast cancers through directly binding to the promoter and modifying the expression of ERα or GATA-3 [21, 23]. Our above results, showing that Notch3 upregulates STAT5A expression, suggest that the Notch3/STAT5A axis modulates the expression of EMT-associated markers and restrains EMT.

**Fig. 5:**
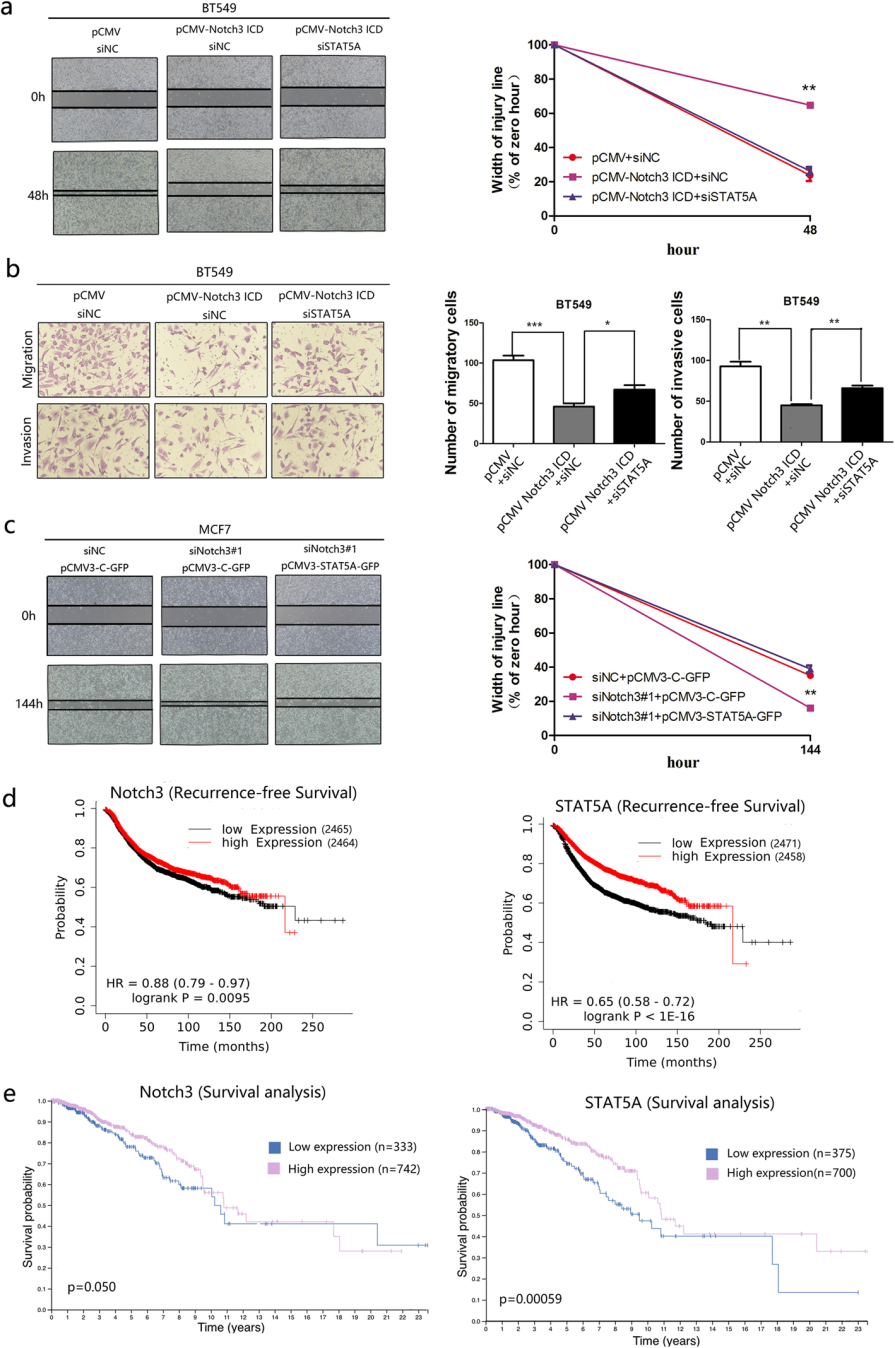
Notch3-mediated suppression of metastasis can be reversed by STAT5A siRNA in breast cancer cells and high Notch3 and STAT5A expression predicts better prognosis in patients with breast cancer. **a** Suppressive effect of Notch3 on wound healing was reversed by suppressing STAT5A expression in BT549 cells. **b** In transwell assays, Notch3-induced decreased mobility of BT549 cells was rescued by knockdown of STAT5A. **c** Enhancement of wound healing by siNotch3#1 was reversed by overexpression of STAT5A in MCF-7 cells. **d** Low expression of Notch3 predicted poor recurrence-free survival in patients with breast cancer. As well as, breast cancer patients with low STAT5A levels showed poor recurrence-free survival. **e** Low expression of Notch3 predicted poor overall survival in patients with breast cancer. And breast cancer patients with low STAT5A levels showed poor overall survival

Rescue experiments showed that overexpression of Notch3 in BT549 cells suppressed cell migration in scratch wound healing assays, but migration could be partially restored through knockdown of the expression of STAT5A (Fig. 5a). Similarly, in transwell assays, Notch3-mediated inhibition of cell migration and invasion of breast cancer cells was partially reversed by suppressing the Notch3 downstream target STAT5A (Fig. 5b). Furthermore, knockdown of Notch3 in MCF-7 cells increased cell migration and wound healing in a scratch wound healing assay, whereas the increased cell migration due to Notch3 knockdown could be partially reversed through overexpression of STAT5A (Fig. 5c).

### Low levels of Notch3 and STAT5A predict poor survival in patients with breast cancer

To elucidate the association of Notch3 and STAT5A with clinical outcomes in patients with breast cancer, Kaplan–Meier plotter, an online database was used to determine the association between mRNA levels of Notch3/STAT5A and clinical endpoints. In all patients, low levels of Notch3 were associated with shortened RFS (*p* = 0.0095, HR = 0.88) (Fig. 5d). As expected, expression of the Notch3-target gene, STAT5A, was also found to be positively associated with the period of recurrence in patients with breast cancer (*p* < 1E-16, HR = 0.65) (Fig. 5d).

Analysis from the Human Protein Atlas revealed that increased Notch3 protein expression was associated with improved survival in patients with breast cancer (*p* = 0.050) (Fig. 5e). Patients with high expression of STAT5A also had longer survival, with a 5-year survival rate of 85%, whereas of those with low expression, 74% had a 5-year survival rate (*p* = 0.00059) (Fig. 5e).

## Discussion

The main cause of cancer-related death is metastasis, and the same is true for breast cancers [31–33]. Although multiple variables, such as genetic/epigenetic modification, population, environment, and daily diet, are reported to be involved in the tumorigenesis and development of breast cancer [34–36], the precise mechanism is still unclear. Importantly, immune suppression and increased oxidative stress in diverse malignancies is predicted to associate with poor survival of patients [37]. In order to develop precision medicine for the treatment of breast cancers, a highly heterogeneous disease, identification of the underlying mechanisms for distant metastasis of breast cancer is very important for extending cancer survival and improving patient quality of life.

STAT family member are a group of transcription factors, regulated and phosphorylated by JAK. Among them, STAT5A was originally reported as a regulator of PRL signaling and milk protein gene expression, and was shown to be essential for mammary development and lactogenesis [38–40]. In breast cancer, the role of STAT5A is complex with a potential dual role in the regulation of malignant mammary epithelium [41]. Wu et al*.* demonstrated that transcriptional STAT5A is a biomarker for prognosis in human breast carcinoma [42]. In the early stages of breast cancer, STAT5A has been shown to promote malignant transformation and enhance tumor growth, whereas in established breast cancer, STAT5A is involved in maintaining the differentiation of mammary epithelium [41]. Ren et al*.* showed that loss of a STAT5A allele reduces the survival of breast cancer cells rather than causes cell proliferation or differentiation of the cancers [43]. Bratthauer et al*.* examined the expression of STAT5A and related prolactin receptor (PRLR) in normal and abnormal breast epithelial tissues and found that STAT5A is overexpressed in over 80% of normal cell nuclei with minimally detected PRLR, whereas the reverse was true for STAT5A and PRLR in breast carcinomas [44]. In luminal breast cancer cell lines, an ERα-STAT5A complex was found, and its functional consequence depended on the precise milieu of the intracellular environment [45]. However, Sultan et al*.* showed that prolactin-activated STAT5A induces formation of an E-cadherin-β-catenin complex in vitro and in xenotransplanted tumors in vivo, leading to inhibition of breast cancer invasion. In addition, JAK2 and STAT5A overexpression cooperatively reverses EMT and promotes differentiation in human breast cancer cells, which explains the suppression of invasion of prolactin-activated STAT5A in mammary cancer cells [46]. Recently, STAT5A/HDAC6/HMGN2 cis-regulatory landscape drived by prolactin was identified as cofactors and therapeuctic targets for synergistic effect in ER-positive breast cancers [47].

Casetti et al*.* demonstrated the contribution of STAT5A to stress protection of chronic myeloid leukemia stem/progenitor cells [48], while Cholez et al*.* provided evidence for the protective role of transcription factor STAT5A against oxidative stress in human leukemic pre-B cells [49]. In current study, STAT5A was predicted to be positively associated with tumor-infiltrating immune cells, including B/T cells, macrophages and dendritic cells. 

The current study shows a role for STAT5A in suppression of invasion, as demonstrated by overexpression or knockdown of STAT5A in different breast cancer cell lines. It has been reported that when EMT is induced, the cells obtain spindle shape and loss of cell–cell contact, otherwise the shape of cells were congregated and circular [50, 51]. Bhatt et al*.* constructed a novel diphenylamine analogs which can induce mesenchymal to epithelial transition in TNBC cells, such as MDA-MB-231 and BT-549, and reported the reversible cellular transformation between cobblestone-like and spindle-like phenotypes asscociated with spindle index [27]. Interestingly, the expression of STAT5A is similar to Notch3 levels in breast cancer cell lines. As expected, enforced Notch3 expression up-regulates the expression of STAT5A in cell lines of the luminal subtype of breast cancer, whereas suppressing Notch3 expression inhibits the expression of STAT5A in triple-negative breast cancer cell lines. As Notch3 has a transcriptional function [52], the promoter region of STAT5A was searched and at least five potential CSL-binding sites were found, indicating the potential of Notch3 to bind to the STAT5A promoter. ChIP assays demonstrated that Notch3 directly binds to the promoter of STAT5A and the direct binding could activate the transcriptional activity of STAT5A, as determined by luciferase reporter gene assay.

**Fig. 6:**
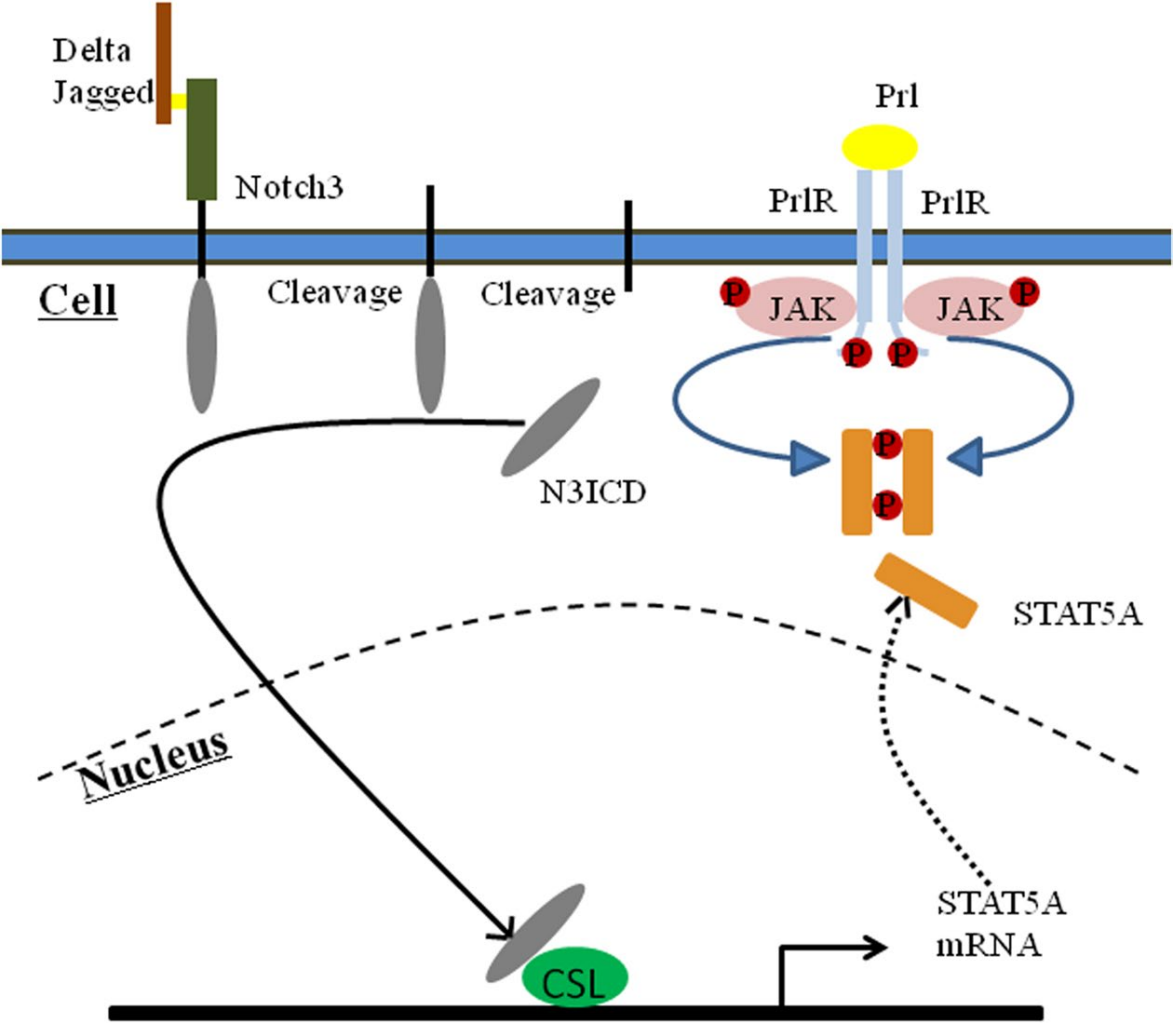
Proposed model of how Notch3 upregulate STAT5A in breast cancer. Notch3 is cleavaged twice to form N3ICD. In the cell nucleus, N3ICD forms a complex with CSL, which initiates transcription of STAT5A. Prolactin(Prl) binding to the receptor(PrlR) results in receptor dimerization and autophosphorylation of the receptor-associated JAK. Then, JAK phosphorylates the receptor, thereby creating docking sites for Src homology 2 (SH2) domain proteins, such as STAT5A. Subsequently, Jak2 phosphorylates the STAT5A to form an active dimer

Previous studies demonstrated anti-metastatic effects of Notch3, a transcription factor, through transcriptional up-regulation of p21[53], estrogen receptor-α (ERα) [21], GATA-3 [23], miR-488/FSCN1 [54] and Kibra-mediated Hippo/YAP signaling [25] in breast cancer epithelial cells. Liang et al*.* revealed that miR-221/222 promotes EMT in breast cancer cell lines through suppressing Notch3 expression [55]. Increasing evidence has shown a regulatory role for Notch3 in breast cancer, and the investigation of potential downstream targets of Notch3 will expand our understanding of the mechanism of breast cancer metastasis. In the current study, STAT5A was first shown to be activated by Notch3 signaling in breast cancer, resulting in suppression of EMT and metastasis of breast cancer cells (Fig. 6).

To investigate the effect of the novel Notch3-STAT5A axis on breast cancer metastasis, rescue experiments were conducted. Ectopic STAT5A knockdown partially restored the migration and invasion of breast cancer cells, following Notch3 overexpression-induced suppression, indicating that Notch3-mediated inhibition of breast cancer metastasis occurs in part through activating STAT5A expression. Tang et al*.* revealed that constitutively active STAT5A enhances both survival and anchorage-independent growth of human mammary carcinoma cells but also suppresses cell motility, as revealed in colony scattering, cell migration, and invasion assays [56]. During mammary cell growth, prolactin suppresses BCL6 expression through a STAT5A-related mechanism, suggesting that loss of prolactin-STAT5A signaling and concomitant increases of BCL6 may constitute a regulatory switch and facilitate undifferentiated histology and poor prognosis of breast cancer [57].

There are some limitations to this study, we found that the expression level of the phosphorylated STAT5 (phospho Y694) is similar to STAT5A levels in breast cancer cell lines. Although the change of STAT5A expression level leads to the change of phosphorylation form, but there's no antibody to distinguish between STAT5A and STAT5B. The promoter region of STAT5A contained 5 CSL-binding elements, but the second and third elements were too close to each other, the corresponding fragment could not be amplified, so the two elements could only be analyzed together. On the other hand, as the expression of STAT5A, p-STAT5 and Notch3 (N3ICD) were highest in MCF-7, a luminal subtype breast cancer cell line and lowest in BT549, a TNBC cell line, the in vitro experiments were conducted in these two subtype breast cancer cell lines to confirm the regulation of Notch3 on the expression of STAT5A and its activation as well. However, the relationship and potential regulatory mechanism between Notch3 and STAT5A was not verified in HER2-positive subtype of breast cancer.

Notably, we also find the expression level of STAT5A to be positively related to Notch3 expression in patients with invasive breast carcinomas. Further exploration demonstrated that low expression of STAT5A and Notch3 both predict poor survival of breast cancer patients, which may be related to the suppressed immune response. Consistently, in breast cancer patients, after examining the protein and transcript levels of STAT5A/5B, Peck et al*.* showed that low STAT5A protein level is an independent marker of poor prognosis in breast cancer, especially in node-negative breast cancer [58]. N-α-Acetyltransferase 10 protein (Naa10p), the catalytic subunit of N-acetyltransferase A, inhibits the metastasis of breast cancer cells by binding STAT5A and decreasing STAT5A-stimulated inhibitor of differentiation 1 (ID1) expression in an acetyltransferase-independent manner [59]. Interestingly, in most human mammary tumors, miR-221/222 are key breast cancer cell proliferation and invasion regulators and exert their effects via post-transcriptional regulation of STAT5A [60], and also suppress Notch3 [55].

In prostate cancer and Bcr-Abl-driven leukemia, researcher sought to identify small molecule inhibitors of Stat5a/b for lead optimization and therapy development [61]. They reported AK-2292 as a potent and selective small-molecule degrader of both STAT5A and STAT5B [62]. Howerer, loss of STAT5A are associated with tumor progression and unfavorable clinical outcomes [58], meaning that drugs targeting STAT5A may caused unexpected outcomes. Meanwhile, current study focused on the tumor suppressor role of STAT5A in luminal and triple-negative subtypes of breast cancer, which is proposed as a novel biomarker for metastasis of breast cancer, evoking the potential therapeutic effect of analogs of STAT5A or N3ICD/STAT5A axis. Peptides can be constructed, and nanomaterials can be used as carriers to achieve targeted therapy of breast cancer.

## Conclusion

In conclusion, STAT5A, an important transcription factor for controlling mammary cell fate, is transcriptionally activated by Notch3 to suppress the metastasis of breast cancer cells, providing novel insight into the complex regulation of EMT and tumor-infiltrating immune cells, and identifying a Notch3/STAT5A pathway as a promising candidate to affect prognosis of breast cancer.

### Supplementary Information

**Additional file 1.**

## Data Availability

The datasets used and/or analyzed during the current study available from the corresponding author on reasonable request.
