# Supplementary material for: Notch3 restricts metastasis of breast cancers through regulation of the JAK/STAT5A signaling pathway
Source: BMC Cancer. 2023 Dec 20;23:1257. doi: 10.1186/s12885-023-11746-w (PMC10734157; doi:10.1186/s12885-023-11746-w)

Fig2f STAT5A

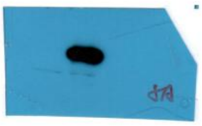

Fig2f P-STAT5

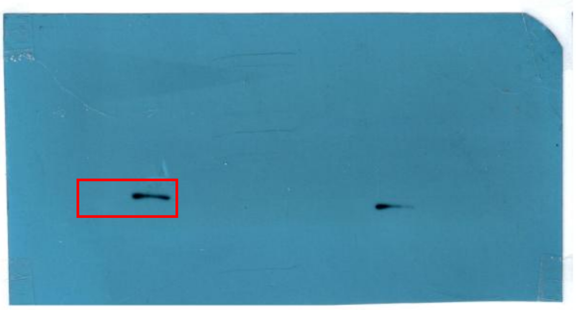

Fig2f Vimentin

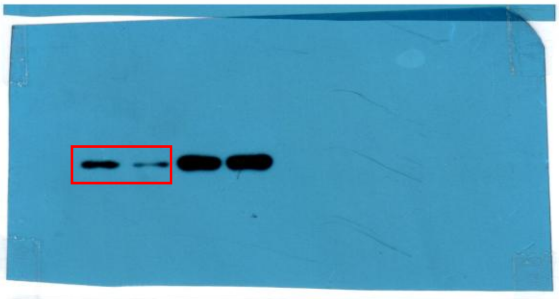

Fig2f GAPDH

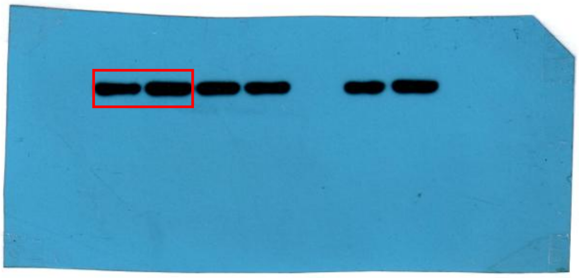

Fig2i STAT5A

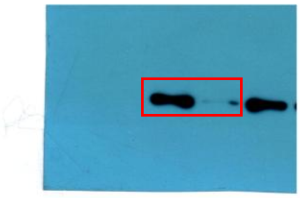

Fig2i E-cadherin

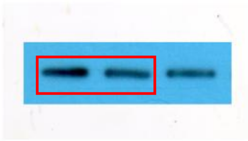

Fig2i GAPDH

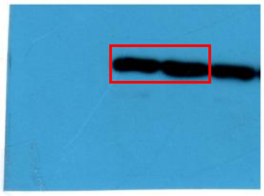

Fig3b N3ICD

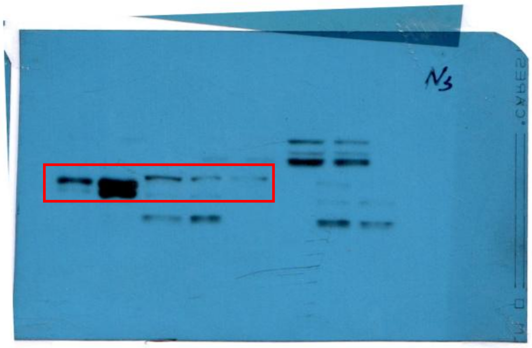

Fig3b STAT5A

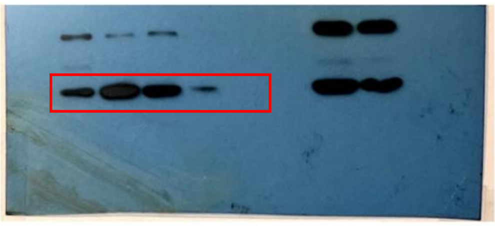

Fig3b P-STAT5

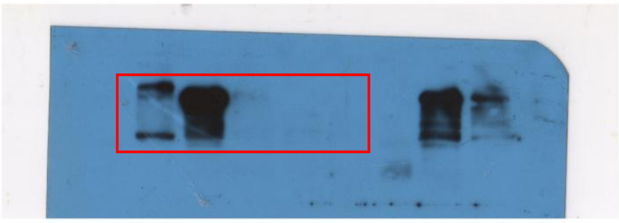

Fig3b GAPDH

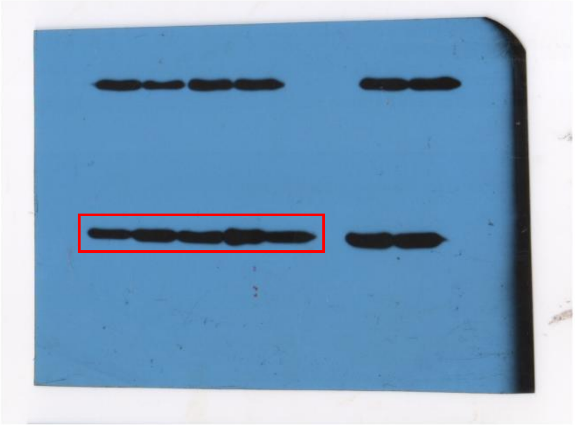

Fig3d N3ICD

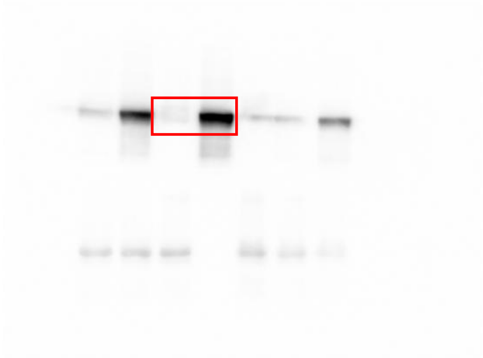

Fig3d STAT5A

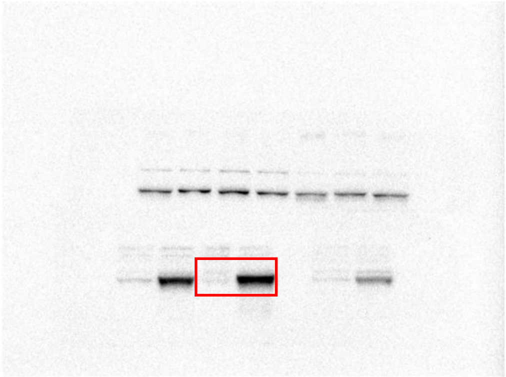

Fig3d P-STAT5

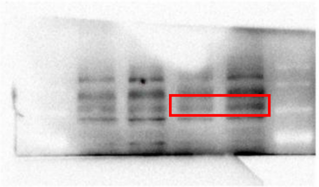

Fig3d β-actin

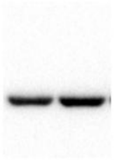

Fig3e N3ICD

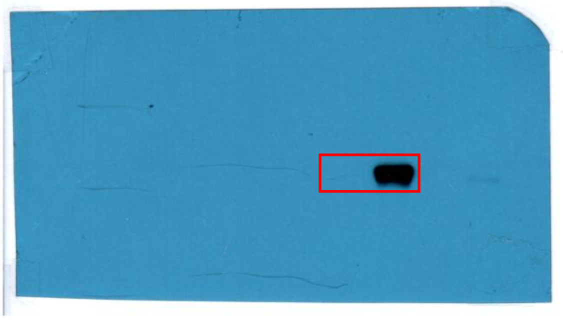

Fig3e STAT5A

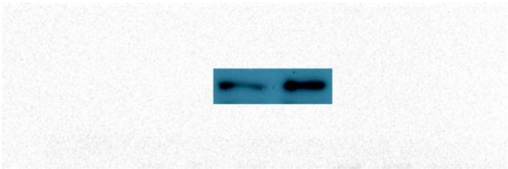

Fig3e P-STAT5

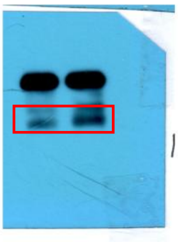

Fig3e β-actin

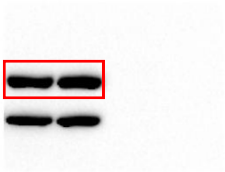

Fig3f N3ICD

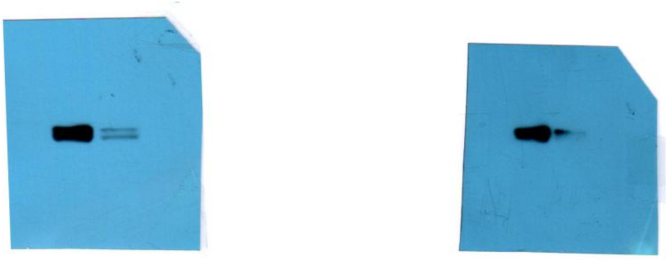

Fig3f STAT5A

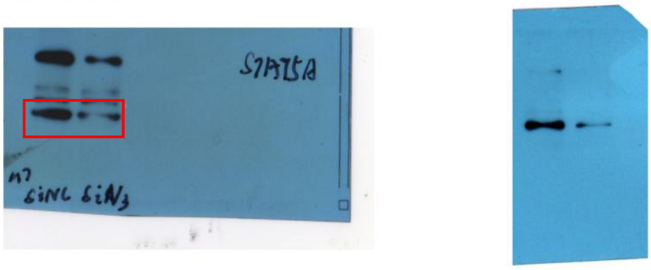

Fig3f P-STAT5

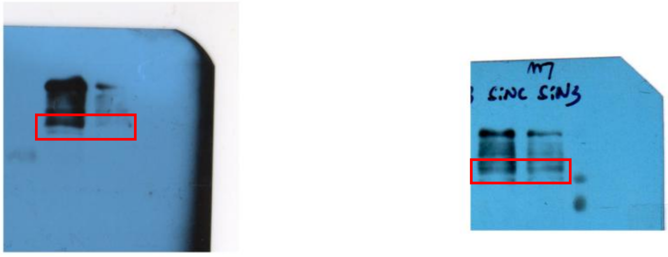

Fig3f GAPDH

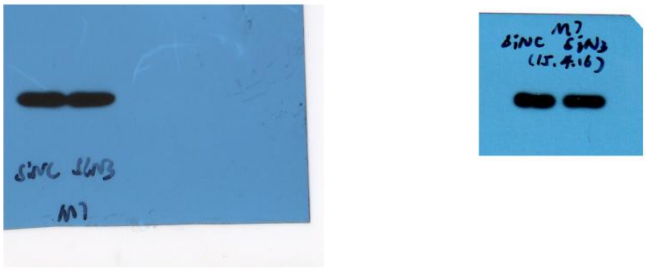

Supplement: Supplementary file 1 — Additional file 1. [file 12885_2023_11746_MOESM1_ESM.pdf]
